# Comprehensive analysis of nutrient partitioning and microbial communities in pear orchards: effects of tree age and spatial heterogeneity

**DOI:** 10.1186/s12870-025-07704-2

**Published:** 2025-12-02

**Authors:** Jiali Peng, Yan Sun, Jingjing Geng, Ziwei Xu, Fangfang Wang, Zhenghong Li, Huibin Wang, Shugang Zhao

**Affiliations:** 1 College of Horticulture, Hebei Agricultural University, Baoding, Hebei 071001 China; 2 Liangshan County Agricultural Technology Extension Center, Liangshan, Shandong 272600 China; 3 College of Life Sciences, Hebei Agricultural University, Baoding, Hebei 071001 China; 4 College of Life Science, Hengshui University, Hengshui, Hebei 053000 China; 5 Pear Industry Technology Engineering Research Center of the Ministry of Education, Baoding, Hebei 071001 China

**Keywords:** Pear, Tree age, Plant nutrition, Soil heterogenicity, Plant–microbiota interactions

## Abstract

**Background:**

Pear tree nutrient requirements vary across growth stages. The spatial distributions of soil nutrients and microbial communities were analyzed to elucidate the nutrient demands at different growth stages, investigate the relationship between soil microorganisms and nutrients, and provide a theoretical basis for fertilization and soil management practices in pear orchards.

**Results:**

Distinct temporal and spatial patterns in nutrient dynamics were observed. With increasing tree age, the leaf calcium (Ca) content initially increased then decreased, peaking at 30.96 g·kg⁻^1^ in 46-year-old trees. The leaf copper (Cu) content progressively increased, reaching its highest concentration (15.16 mg·kg⁻^1^) in trees (> 100 years). The relative abundances of key bacterial phyla, *Actinobacteria* and *Acidobacteria*, reached their maxima (19.88% and 20.4%, respectively) in 4-year-old orchards before slightly declining. Spatial analysis of mature orchards revealed that the soil phosphorus (P) and potassium (K) contents decreased with increasing distance from the tree trunk, whereas the boron (B), zinc (Zn), and manganese (Mn) contents increased. Comparative analysis with adjacent long-term unplanted soils revealed that pear tree cultivation significantly depleted soil Ca, magnesium (Mg), iron (Fe), and Mn, whereas Cu and Zn levels increased, suggesting a high tree demand for the former group and an application rate of Cu and Zn fertilizers exceeding the tree absorption capacity. Within tree tissues, the nitrogen (N) and P contents were highest in 1- and 2-year-old branches, whereas Ca, Mg, Fe, B, and Mn accumulated predominantly in perennial old roots. Significant positive correlations were identified between several leaf and soil elements. Furthermore, soil nutrient availability was linked to microbial diversity: soil P and Zn contents were positively correlated with the bacterial aroma index. Soil Mg, Cu, and Zn were positively correlated with the bacterial ACE index; and the soil N content was positively correlated with the fungal Simpson index.

**Conclusion:**

Soil microbial communities in pear orchards are associated with P, Ca, and Zn. Nutrient elements in pear trees such as Ca, Mn, B, Mg, and Fe, which are difficult to transport and tend to accumulate in root tissues; thus, foliar application is recommended for their supplementation.

**Supplementary Information:**

The online version contains supplementary material available at 10.1186/s12870-025-07704-2.

## Background

Pear is the main cultivated fruit tree in China and one of the most widely planted fruit trees in China [1, 2]. Leaf and branch nutrition efficiently reflects a tree's soil nutrient use while simultaneously serving as a direct indicator of its developmental status [3, 4]. Consequently, performing nutritional assessments on leaves and branches can provide an effective means of guiding fruit tree fertilization [5]. As perennial fruit trees, pears present challenges for thorough orchard soil tillage, which readily leads to uneven fertilizer distribution. Therefore, data on leaf nutrient status must be incorporated into the design of formulated fertilization strategies [6, 7]. Most studies have confirmed that both the soil nutrient content and leaf nutrition affect tree growth and development and fruit quality [8]. Compared with medium- and low-quality orchards, orchards that produce superior-quality fruit generally have higher soil organic matter, nitrogen (N), phosphorus (P), and potassium (K) contents [9, 10]. Notably, compared with those in other orchards, potassium levels are significantly higher [11]. In terms of yield, the soil organic matter, available phosphorus, and available iron contents in high-yield pear orchards are significantly greater than those in low- and medium-yield pear orchards [12, 13]. Similarly, the contents of nutrient elements in pear leaves are closely related to the yield. For example, the dynamic equilibrium of phosphorus and iron jointly influences yield by regulating root morphology and elemental uptake efficiency [14, 15]. Nutrient elements in leaves and soil also vary with tree age. For instance, as tree age increases, microbial nitrogen accumulation initially decreases and then increases in young (8 years) and old (76 years) orchards compared to mature orchards (36 years) [16]. Nevertheless, the nutrient content in trees is nonlinearly related to tree age. Young trees demonstrate a more pronounced demand for nitrogen, but as trees mature, their requirements for different nutrients become inconsistent because of the influence of reproductive growth [17, 18]. Therefore, clarifying the relationship between soil nutrition and tree nutrition in pear orchards is helpful for guiding formulated fertilization.

Microbes are an extremely abundant group of organisms in orchard soils and have become the focus of research in recent years [19, 20]. Different fertilization regimes can affect the population, quantity and activity of soil microbes, which can lead to differences in soil biological fertility and simultaneously affect soil structure, fertility and productivity [21, 22]. During the renovation of aging orchards, soil fumigation combined with compost application alters the soil microbial community, and fungi and bacteria in the rhizosphere may influence tree growth and yield [23]. Applying an appropriate manure ratio significantly enhances soil enzyme activity and shapes distinct bacterial community patterns [24]. To date, few studies have investigated the relationships between the types and contents of soil nutrients in pear orchards and microbial communities.

Hebei Province is among the main pear-producing areas in China, where most of the pear-producing areas are pear orchards with trees older than 20 years [25]. Usually, in the pursuit of high yield in aging pear orchards, habitual fertilization causes unbalanced soil nutrition, which also causes unbalanced tree nutrition, reducing fruit quality [26]. Therefore, in this study, the leaf nutrition, soil nutrition and soil microbial communities at different tree ages were analyzed to determine the distribution patterns of nutrient elements in the soil and trees and the changes in the soil microbial communities with increasing tree age. The changes in the nutrient element needs of trees provide a theoretical basis for precision fertilization to improve fruit quality.

## Materials and methods

### Experimental site and design

The experimental site was located in Dongzhangkou village, Xinji city, Hebei Province (115°8′2.112″, 37°51′33.876″), which is the core production area of Huangguan pear in Hebei Province. The experimental site was located on the ancient course of the Hutuo River and has sandy loam soil and a continental temperate monsoon climate, with an average annual temperature of 12.1 °C, a frost-free period of 188 days, and an annual average precipitation of 488.2 mm. The soil of this experimental orchard is sandy loam, and all the parks followed unified management in terms of the base fertilizer and top-dressing links. The base fertilizer was applied in late October (after fruit picking), and 15 kg of decomposed cow manure and 1.0 kg of nitrogen, phosphorus and potassium compound fertilizer (N-P₂O₅-K₂O: 15–15–15) were applied in a circular furrow under the canopy projection at a depth of 30–40 cm. Top dressing is carried out in two parts: the first time in early March (before budding), 0.3 kg of urea was applied to each plant; in mid-May (young fruit expansion stage), and 0.5 kg of potassium sulfate was applied to per hole.

All test material was Huangguan pear (*Pyrus bretschneideri* 'Huangguan'), with *Pyrus betulifolia* used as rootstock, which was planted in the same orchard of different plots. The ages of the trees in the five plots were 4 years (y), 25 y, 46 y, 65 y and more than 100 years, and the management levels were the same. The management of fertilization and irrigation was consistent in each plot. Soil organic matter in different plots was 10.7 g·kg^−1^ (4 y), 12.5 g·kg^−1^ (25 y), 14.3 g·kg^−1^ (46 y), 13.9 g·kg^−1^ (65 y), and 15.4 g·kg^−1^ (100 y), respectively. Soil pH of different plots was 7.81(4 y), 7.93(25 y), 7.76(46 y), 7.82(65 y), and 7.73(100 y), respectively. The planting density was 1 m × 4 m for the 4-year-old tree plot and 3 m × 5 m for the other plots.

In early April, soil samples were collected from each pear orchard in the park according to its area and shape via according to the five-point method, and each point was divided into two soil layers (0–20 cm and 20–50 cm). The sampling point was the rhizosphere soil of the pear trees after the mulch was removed. This process was repeated three times. The samples were mixed evenly, and the soil was removed. The humus and impurities in the soil were sieved (40 mesh, i.e., 0.43 mm), loaded into sterilized 10 mL centrifuge tubes and quickly refrigerated for soil nutrient and microbial analysis, respectively.

During the growing season, 10 pear trees whose growth was uniform were selected. Good leaves without mechanical damage were newly obtained from the middle and upper parts of each tree in the four directions (east, south, west, and north). 20 leaves from each tree were selected.

**Fig. 1:**
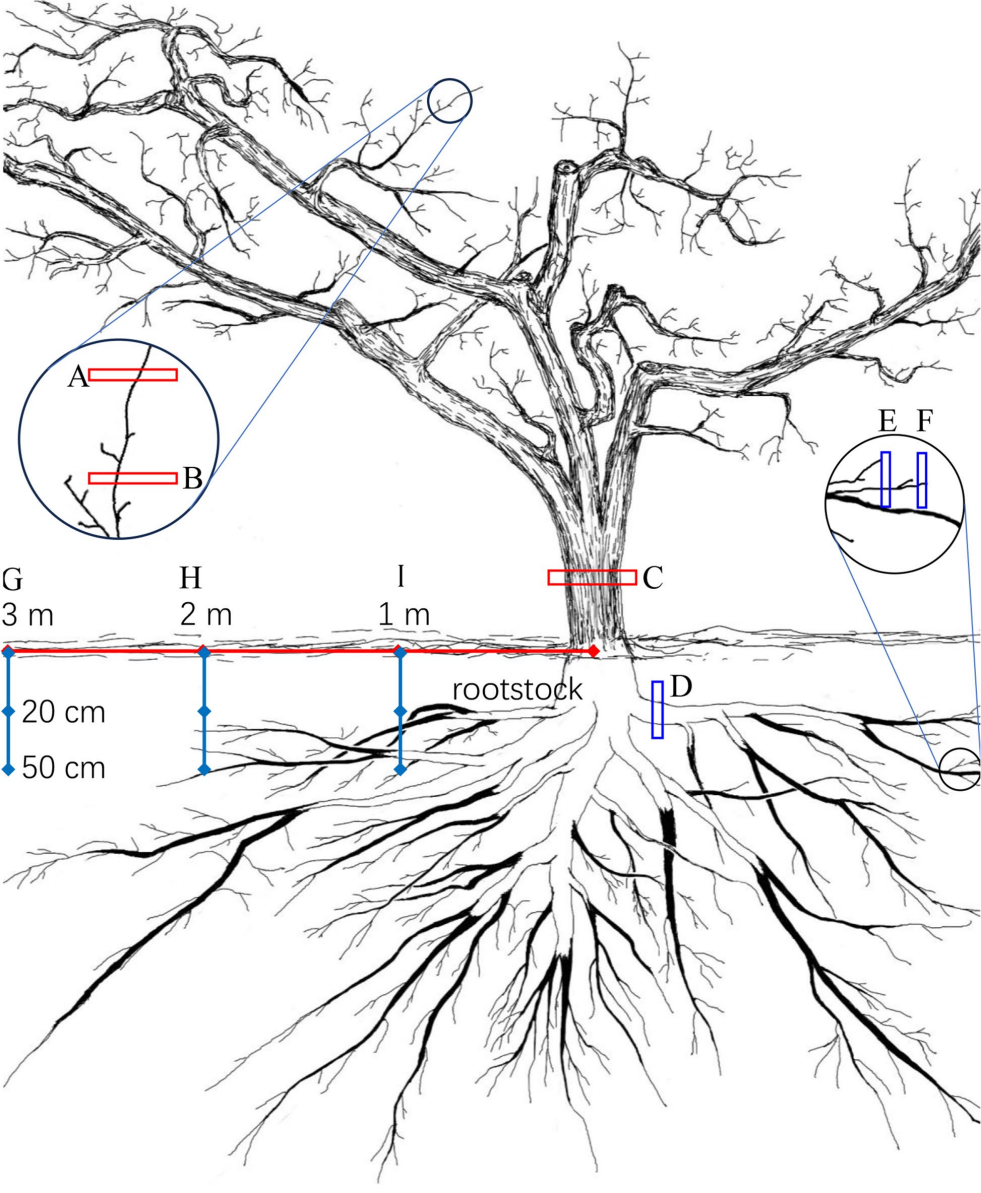
Soil sampling at different locations and collection of different branches . **A** One-year-old branches (ABs). **B** Two-year-old branches (BBs). **C** Trunk (65 years old). **D** 10-year-old roots (OR10y). **E** Two-year-old roots (OR2y). **F **One -year-old roots (FRs). **G** Sampling point 3 m from trunk. **H** Sampling point 2 m from trunk. **I** Sampling point 1 m from trunk

During the dormant season (February 2023), five representative mature pear trees (65 years old, average canopy size 2.8 m × 5.2 m) that were growing well and whose growth was consistent were selected. The trunks (growth cones were used to sample the xylem (X65y) and phloem (P65y)), 10-year-old roots (OR10y), one-year-old branches (AB), two-year-old branches (BB), one-year-old roots (FR) and two-year-old roots (OR2y) were evaluated. Moreover, two soil layers, 0–20 cm and 20–50 cm, were collected with a soil remover at locations 1 m, 2 m and 3 m away from the trunk, respectively (Fig. 1). Additionally, soil samples from a nearby field with no history of pear cultivation were collected from four positions (east, south, west, north) at the same two depths (0–20 cm and 20–50 cm) as controls.

### Soil and leaf analysis

The soil samples were naturally air-dried, ground and sieved. The soil samples were subsequently selected and digested via the quartering method to determine their nutrient element contents. The leaves were subsequently washed with tap water, detergent, 0.2% hydrochloric acid or deionized water, fixed at 105 °C, dried at 80 °C, crushed and stored for future use [27]. A 0.1-g sample was weighed and transferred to a digestion tube, after which 6 mL of a mixed solution of nitric acid:perchloric acid (5:1) was added. Digestion began at 110 °C, and the temperature was gradually increased to 180 °C. The samples were digested for 4 h and then diluted to a final volume of 50 mL with ultrapure water. Eight nutritional elements were measured using inductively coupled plasma‒optical emission spectrometry (Prodigy Plus, Teledyne Leeman Labs, USA). The optimized ICP‒OES instrument conditions included an RF power of 1150 W, an auxiliary gas flow rate of 0.8 L/min, and a nebulizer gas flow rate of 0.95 L/min. The wavelength lines for each element were K 404.721 nm, Ca 183.034 nm, Mg 285.204 nm, Cu 324.754 nm, Fe 238.204 nm, B 249.677 nm, Zn 213.856 nm, and Mn 259.373 nm. The total nitrogen (N) and total phosphorus (P) contents were determined via AutoAnalyzer 3 (SEAL Analytical, Germany). Each sample was tested in triplicate.

### Sequencing analysis of soil microbes

Rhizosphere soil samples collected from trees of different ages were sent to Majorbio Bio-Pharm Technology Co., Ltd. (Shanghai, China) for sequencing analysis on the Illumina PE300 platform. Total microbial genomic DNA was extracted from soil samples from different plots using an E.Z.N.A.® soil DNA Kit (Omega Biotek, Norcross, GA, U.S.) according to the manufacturer’s instructions. The hypervariable region V3-V4 of the bacterial 16S rRNA gene was amplified with the primer pairs 338 F (5'-ACTCCTACGGGAGGCAGCAG-3') and 806R (5'-GGACTACHVGGGTWTCTAAT-3') by a T100 Thermal Cycler PCR thermocycler (Bio-Rad, USA). The PCR mixture included 4 μL of 5 × Fast Pfu buffer, 2 μL of 2.5 mM dNTPs, 0.8 μL of each primer (5 μM), 0.4 μL of Fast Pfu polymerase, 10 ng of template DNA, and ddH_2_O to a final volume of 20 µL. The PCR amplification cycling conditions were as follows: initial denaturation at 95 °C for 3 min; 27 cycles of denaturation at 95 °C for 30 s, annealing at 55 °C for 30 s and extension at 72 °C for 45 s; and a single extension at 72 °C for 10 min, ending at 4 °C. The PCR product was extracted from a 2% agarose gel and purified using a PCR Clean-Up Kit (YuHua, Shanghai, China) according to the manufacturer’s instructions and quantified using a Qubit 4.0 (Thermo Fisher Scientific, USA). Purified amplicons were pooled in equimolar amounts and paired-end sequenced on an Illumina PE300 (Illumina, San Diego, USA) according to the standard protocols by Majorbio Bio-Pharm Technology Co. Ltd. (Shanghai, China).

### Data analysis

Microsoft Excel 2019 software and Meiji Biotechnology were used for data analysis, and SPSS 26.0 was used for significance analysis. Bioinformatic analysis of the soil/gut microbiota was carried out using the Majorbio Cloud platform (https://cloud.majorbio.com). Detailed explanations of the calculation methods for the Shannon index, Simpson index, ACE index, and Chao index can be found in Additional file 1.

## Results

### Nutrient element contents of pear tree leave at different ages

**Fig. 2:**
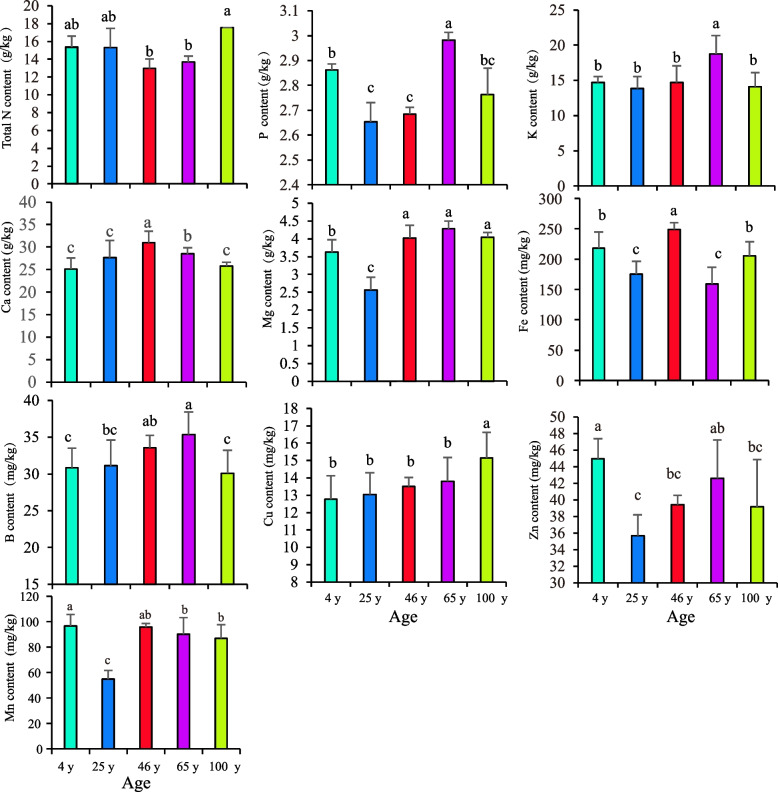
Nutrient content in leaves at different tree ages. Note: Different lowercase letters indicate significant differences among treatments at *P* < 0.05

To investigate the relationships between pear trees and soil in terms of nutrient elements, we first tested the concentrations of the elements in the pear leaves. As shown in Fig. 2, significant differences in macroelements and microelements were detected in the leaves of pear plants planted in different-aged orchards. The concentrations of Zn and Mn in the leaves of the pear orchard at the age of 4-year-old were 44.98 mg·kg^−1^ and 96.56 mg·kg^−1^, respectively. The peak concentrations of Ca and Fe occurred in the leaves of the 46-year-old orchard. The contents of P, K, Mg, and B in the leaves of the 65-year-old pear orchard were the highest at 2.98 g·kg^−1^, 18.75 g·kg^−1^, 4.29 g·kg^−1^and 35.34 mg·kg^−1^, respectively. The contents of K and P were significantly greater than those at other tree ages; the B content initially increased but then decreased with tree age. The highest concentrations of N (17.56 g·kg⁻^1^) and Cu (15.16 mg·kg⁻^1^) were detected in the leaves of the 100-year-old pear orchard, with Cu concentrations increasing significantly with age. The 25-year-old orchard had the lowest leaf P (2.65 g·kg⁻^1^), K (13.85 g·kg⁻^1^), Mg (2.56 g·kg⁻^1^), Zn (35.70 mg·kg⁻^1^), and Mn (54.85 mg·kg⁻^1^) concentrations, with the Mg and Mn concentrations being significantly lower than those at other ages. The lowest leaf N concentration (12.96 g·kg⁻^1^) occurred in the 46-year-old orchard, whereas the lowest Fe concentration (159.06 mg·kg⁻^1^) occurred in the 65-year-old orchard. Beyond 65 years of age, multiple leaf nutrients, including P, K, Ca, B, and Zn, generally decreased, indicating weakened tree growth vigor.

### Differences in soil nutrients among plots with different tree ages

Significant differences in soil nutrient accumulation were also observed among pear orchards of varying ages (Table 1). At the 0–20 cm depth, K and Mg in the 4-year-old pear orchard were significantly lower than those in the other age groups, measuring 6.35 g·kg⁻^1^ and 9.38 g·kg⁻^1^, respectively. The 25-year-old orchard had the lowest Cu and Zn contents (21.85 mg·kg⁻^1^ and 48.16 mg·kg⁻^1^, respectively), whereas the highest soil N content (1.91 g·kg⁻^1^) was recorded in the 46-year-old orchard. In the 65-year-old orchard, the K, Ca, Mg, Fe, B, Mn, and Cu contents were the highest, at 8.44 g·kg⁻^1^, 37.72 g·kg⁻^1^, 12.38 g·kg⁻^1^, 102.45 mg·kg⁻^1^, 545.50 mg·kg⁻^1^, and 68.77 mg·kg⁻^1^, respectively, with the concentrations of Ca, Fe, B, and Mn being significantly greater than those in the other age groups. In contrast, the lowest N and P contents were observed in the 65-year-old orchard, at 0.87 g·kg⁻^1^ and 3.29 g·kg⁻^1^, respectively. The 100-year-old orchard had the highest P and Zn concentrations (10.69 g·kg⁻^1^ and 73.76 mg·kg⁻^1^, respectively), while the Fe, Ca, B, and Mn concentrations were the lowest (13.46 mg·kg⁻^1^, 27.43 g·kg⁻^1^, 73.64 mg·kg⁻^1^, and 330.48 mg·kg⁻^1^, respectively). The Zn content increased with tree ages, and the P content was significantly greater than that at other ages.

**Table 1:** Analysis of soil nutrient status in pear orchards of different ages

Element	Depth(cm)	4 years	25 years	46 years	65 years	100 years
N (g·kg^−1^)	0–20	1.76 ± 0.22 a	1.20 ± 0.14 b	1.91 ± 0.17 a	0.87 ± 0.24 b	0.92 ± 0.08 b
	20–50	1.42 ± 0.03 b	1.14 ± 0.05 c	1.82 ± 0.15 a	0.83 ± 0.24 c	2.10 ± 0.17 a
P (g·kg^−1^)	0–20	3.83 ± 0.72 c	3.59 ± 0.90 cd	6.37 ± 0.91 b	3.29 ± 0.38 d	10.69 ± 0.21 a
	20–50	7.07 ± 0.79 a	3.74 ± 0.88 b	6.71 ± 0.35 a	3.83 ± 0.34 b	6.89 ± 0.41 a
K (g·kg^−1^)	0–20	6.35 ± 0.42 c	7.50 ± 0.72 b	7.66 ± 1.35 ab	8.44 ± 1.20 a	7.45 ± 0.18 b
	20–50	5.86 ± 1.01 c	6.09 ± 1.60 c	9.77 ± 1.64 a	8.26 ± 0.74 b	6.86 ± 0.23 c
Ca (g·kg^−1^)	0–20	28.15 ± 2.89 b	34.86 ± 1.11b	29.35 ± 2.99 b	37.72 ± 2.99 a	27.43 ± 1.16 b
	20–50	35.73 ± 0.50 a	28.36 ± 2.14 b	28.11 ± 0.33 b	35.53 ± 0.26 a	35.53 ± 0.20 a
Mg (g·kg^−1^)	0–20	9.38 ± 1.09 c	10.86 ± 0.35 b	11.04 ± 1.23 b	12.38 ± 1.47 a	11.40 ± 0.75 ab
	20–50	10.75 ± 1.57 b	8.63 ± 0.85 d	10.89 ± 0.12 b	12.60 ± 0.20 a	9.74 ± 0.14 c
Fe (g·kg^−1^)	0–20	14.62 ± 0.55 b	13.96 ± 0.08 c	15.15 ± 1.18 b	16.62 ± 1.05 a	13.46 ± 0.27 c
	20–50	13.79 ± 1.18 b	7.70 ± 0.07 c	15.03 ± 0.45 a	15.21 ± 0.31 a	13.55 ± 0.07 b
B (mg·kg^−1^)	0–20	85.75 ± 7.83 b	83.13 ± 4.87 bc	89.99 ± 14.67 b	102.45 ± 13.77 a	73.64 ± 2.28 c
	20–50	72.46 ± 11.09 b	31.09 ± 0.38 c	89.76 ± 7.80 a	87.33 ± 4.14 a	78.35 ± 3.97 b
Cu (mg·kg^−1^)	0–20	26.26 ± 1.32 b	21.85 ± 5.79 c	64.38 ± 3.02 a	68.77 ± 2.59 a	61.43 ± 1.67 a
	20–50	22.75 ± 2.78 c	14.55 ± 6.14 c	43.65 ± 1.94 b	43.13 ± 0.70b	47.23 ± 0.51 a
Zn (mg·kg^−1^)	0–20	51.69 ± 2.04 c	48.16 ± 2.14 d	60.39 ± 3.52 b	59.66 ± 5.51 b	73.76 ± 1.94 a
	20–50	48.10 ± 5.46 bc	22.74 ± 0.09 d	54.08 ± 2.92 a	51.41 ± 2.68 ab	43.31 ± 1.09 c
Mn (mg·kg^−1^)	0–20	386.36 ± 23.83 d	397.55 ± 7.01 c	409.07 ± 29.24 b	545.50 ± 8.04 a	330.48 ± 10.09 d
	20–50	325.27 ± 27.18 c	140.77 ± 1.20 d	391.97 ± 17.55 b	410.56 ± 8.99 a	316.51 ± 3.42 c

The letters in the table denote significant differences at the 0.05 level, as determined by Duncan’s multiple range test, and different letters after the same row of data indicate significant differences. The same applies below

At a soil depth of 20–50 cm, the 4-year-old pear orchard had the highest P and Ca contents (7.07 and 35.73 g·kg⁻^1^, respectively) and the lowest K content (5.86 g·kg⁻^1^). The 25-year-old orchard presented the lowest levels of N, P, Mg, Fe, B, Cu, Zn, and Mn, with values of 1.14 g·kg⁻^1^, 3.74 g·kg⁻^1^, 8.63 g·kg⁻^1^, 31.09 mg·kg⁻^1^, 14.55 mg·kg⁻^1^, 22.74 mg·kg⁻^1^, and 140.77 mg·kg⁻^1^, respectively. In the 46-year-old orchard, the contents of K, B, and Zn were the highest (9.77 g·kg⁻^1^, 89.76 mg·kg⁻^1^, and 54.08 mg·kg⁻^1^, respectively). The 65-year-old orchard had the highest Mg, Fe, and Mn contents (12.60 g·kg⁻^1^, 15.21 g·kg⁻^1^, and 410.56 mg·kg⁻^1^, respectively), whereas the 100-year-old orchard had the lowest Mg (9.74 g·kg^−1^) and Mn (316.51 mg·kg^−1^) contents.

These results indicate that when tree age is less than or equal to 25 years, most nutrient elements in different soil layers are present at relatively low or moderate levels. At 46 and 65 years of age, the nutrient element concentrations in the soil layers increase, whereas at 100 years, the nutrient element content varies. It is speculated that when tree age exceeds 46 years, reduced root absorption capacity leads to increased accumulation of nutrients.

### Soil microbial diversity in pear orchards of different ages

The higher the Shannon index, Chao index, and ACE index and the lower the Simpson index are, the greater the diversity and evenness of the microbial community, and the richer the community [28, 29]. As shown in Table 2, the Shannon index, ACE index, and Chao index of the soil with an age of more than 100-year-old were the highest, indicating that the bacterial communities with an age of more than 100-year-old had the highest richness. The Chao index increased with increasing tree age. The richness of the bacterial communities in the soil of the aged pear orchard did not decrease with increasing tree age but tended to increase.

**Table 2:** Diversity indices of soil bacterial diversity in pear orchards of different ages

Age	Shannon index	Simpson index	ACE index	Chao index
4 years	4.86 ± 0.07 ab	0.02 ± 0.01 a	652.31 ± 16.59 c	670.38 ± 7.30 b
25 years	4.91 ± 0.04 a	0.02 ± 0.01 a	720.54 ± 10.87 b	737.06 ± 26.53 a
46 years	5.03 ± 0.09 a	0.02 ± 0.01 a	732.41 ± 12.15 b	740.977 ± 14.18 a
65 years	4.77 ± 0.07 b	0.02 ± 0.01 a	726.72 ± 11.19 b	743.88 ± 12.89 a
100 years	5.04 ± 0.03 a	0.02 ± 0.01 a	748.41 ± 8.78 a	759.00 ± 13.54 a

As shown in Table 3, the Shannon index, ACE index and Chao index of the 4-year-old soil were the highest, indicating that the fungal community at this age class exhibited the greatest diversity; the Shannon index, ACE index and Chao index of the 25-year-old soil were the lowest, indicating that the fungal community in the 25-year-old soil was the oldest. The richness and evenness of the trees were lower. The Shannon index was 16.54% lower than that of the trees aged 4 years, 10.92% lower than that of the trees aged 46 years, 12.85% lower than that of the trees aged 65 years, and 12.30% lower than that of the trees aged more than 100 years.

**Table 3:** Diversity indices of soil fungi in pear orchards of different ages

Age	Shannon index	Simpson index	ACE index	Chao index
4 years	3.40 ± 0.56 a	0.15 ± 0.08 b	404.07 ± 11.37 a	401.71 ± 10.82 a
25 years	1.734 ± 1.01 b	0.17 ± 0.10 b	262.21 ± 10.63 c	263.80 ± 11.92 c
46 years	2.83 ± 0.42 a	0.51 ± 0.23 a	336.10 ± 46.94 b	319.67 ± 77.11 b
65 years	3.02 ± 0.06 a	0.10 ± 0.01 b	309.15 ± 18.08 b	310.54 ± 10.64 b
100 years	2.97 ± 0.17 a	0.18 ± 0.04 b	326.45 ± 7.20 b	330.28 ± 6.16 b

**Fig. 3:**
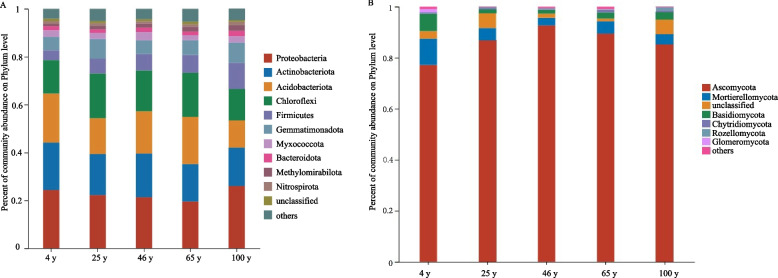
Bacterial and fungal community abundance percentages at the phylum level in soils of different tree ages. **A ** Bacteria **B**. Fungi

To elucidate the dynamics of the soil microbial communities at different tree ages, clustered OTU representative sequences were annotated taxonomically. With respect to bacteria, 6111 OTUs from soil samples of various ages belonged to 46 phyla, 130 classes, 332 orders, 533 families, 1025 genera and 2070 species (Additional file S2). At the phylum level, the dominant soil bacteria at different tree ages were Proteobacteria, Actinobacteria, Acidobacteria, and Chloroflexi, with the total relative average abundance reaching more than 11% (Fig. 3). At the genus level, *Candidatus Koribacter*, *norank_f_norank_o_Acidobacteriales*, *norank_f_norank_o_norank_c_AT-s3-28*, *Abditibacterium*, and *norank_f_norank_o_AMGG11* were the 5 most abundant bacterial genera. However, the dominant microbial communities in the soil differed among trees of varying ages. The relative abundances of *Actinobacteria* and *Acidobacteria* peaked in 4-year-old trees (19.88% and 20.4%, respectively), indicating that the soil was relatively barren in terms of nutrition or that the refractory organic matter was mostly in the undegraded state in the young stage. The abundance of Proteobacteria was the highest in the phylum at 100 years old. The relative abundance was the highest at 26.00% at the tree age of 100 years, indicating that after long-term pear tree planting and nutrient accumulation, such as fertilization, the soil was in the eutrophic rhizosphere. The relative abundance of Chloroformis at the age of 25 years was the highest at 18.67%. With the exception of those aged 65 years, the relative abundance of Proteobacteria in trees aged 4 years, 25 years, and 46 years and above 100 years was significantly greater than that of the other bacterial phyla, while there was no significant difference among the other bacterial phyla.

A total of 2568 fungal OTUs in the soil samples of different ages belonged to 14 phyla, 46 classes, 105 orders, 231 families, 494 genera, and 850 species (Additional file S3). Among them, Ascomycota and Mortierellomycota were the dominant phyla, and *Lasiodiplodia*, *Cladosporium*, *Leptodiscella*, *Archaeorhizomyces*, and *Aplosporella* were the top 5 dominant genera. The relative abundance of Ascomycota tended to increase with increasing tree age, reaching a maximum of 92.7% at 46 years. The relative abundance gradually decreased. The variation range of the Mycotic phylum was 2.94% to 10.31%. The relative abundances of the 4-year-old trees and the phylum were significantly greater than those at other ages, accounting for 10.31%, and there was no significant difference among the other tree ages (Fig. 3).

### Distribution of nutrient elements in different types of pear branches (65 years old)

**Fig. 4:**
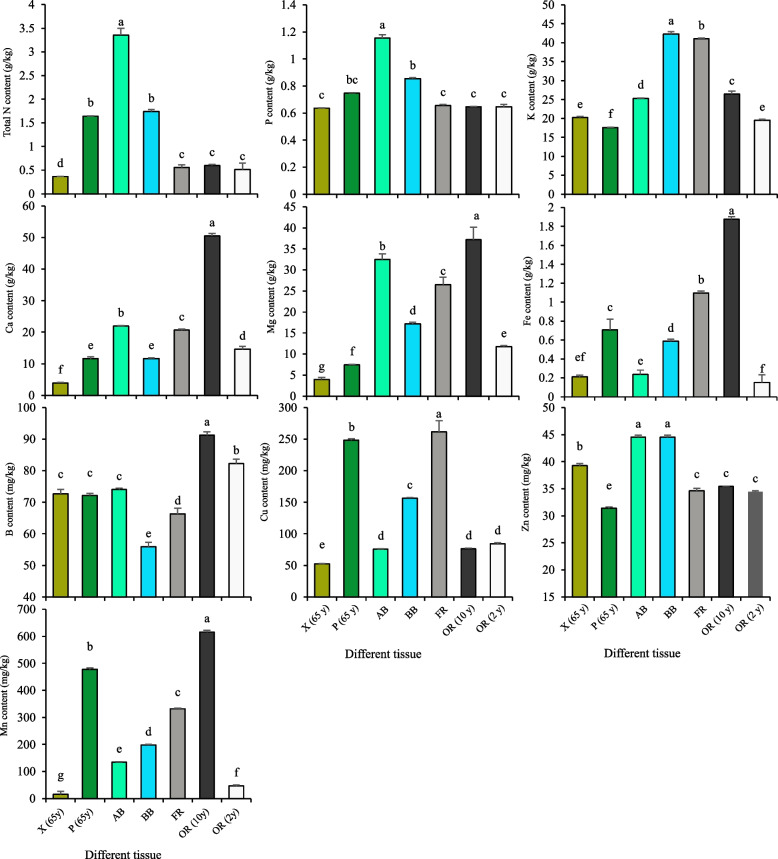
The distribution of nutrient elements in different parts of the branches. Note: Different lowercase letters indicate significant differences among treatments at *P* < 0.05.One-year-old branches (AB), Two-year-old branches (BB), Stem xylem (X(65y)), Stem phloem (P(65y)), 10-year-old roots (OR10y), One -year-old roots (FR), Two-year-old roots (OR2y)

Nutrient element content varies significantly across different branch ages and tissues within the tree, with mobile elements accumulating predominantly in the underground parts. As shown in Fig. 4, N and P were distributed mainly in one-year-old branches, two-year-old branches and the phloem of the trunk (65-year-old), and the N content of the one-year-old branches was 3.35 g·kg^−1^. The P content was 1.15 g·kg^−1^, whereas the N content in the trunk xylem was the lowest at 0.37 g·kg^−1^. K was distributed mainly in biennial shoots and annual fibrous roots, with the K content in biennial shoots being significantly greater than that in other parts (42.26 g·kg^−1^), whereas the K content in the trunk phloem was lowest. The Ca content in the 10-year-old roots was significantly greater than that in the other locations, reaching 50.55 g·kg^−1^, while the accumulation of Ca in the trunk xylem was the lowest. The distribution pattern of Mg was consistent with that of Ca. In both cases, the accumulation of Mg was greatest in 10-year-old roots at 37.15 g·kg^−1^, followed by one-year-old branches and annual fibrous roots, whereas the accumulation in trunk xylem was lowest at 3.96 g kg^−1^. The distribution pattern of Fe was similar to that of Ca and Mg. It accumulated the most in 10-year-old roots, followed by annual fibrous roots, whereas it accumulated less in two-year-old branches, trunk xylem and biennial fibrous roots. Element B was more distributed in 10-year-old roots and biennial roots, whereas it was least distributed in annual fibrous roots and biennial shoots, with the B content in biennial shoots being only 90 mg·kg^−1^. Cu accumulated the most in the 10-year-old roots at 262.0 mg·kg^−1^, whereas it accumulated the least in the trunk xylem at 52.18 mg·kg^−1^. Zn accumulated the most in annual and biennial shoots, whereas it was least distributed in the phloem of the trunk (31.4 mg·kg^−1^). The distribution patterns of Mn and Fe in each tissue were consistent. The accumulation in the 10-year-old roots was greatest at 616.17 mg·kg^−1^, and the distribution in the trunk xylem was the lowest at 15.17 mg·kg^−1^.

### Spatial distribution of soil nutrient elements in pear orchards

**Fig. 5:**
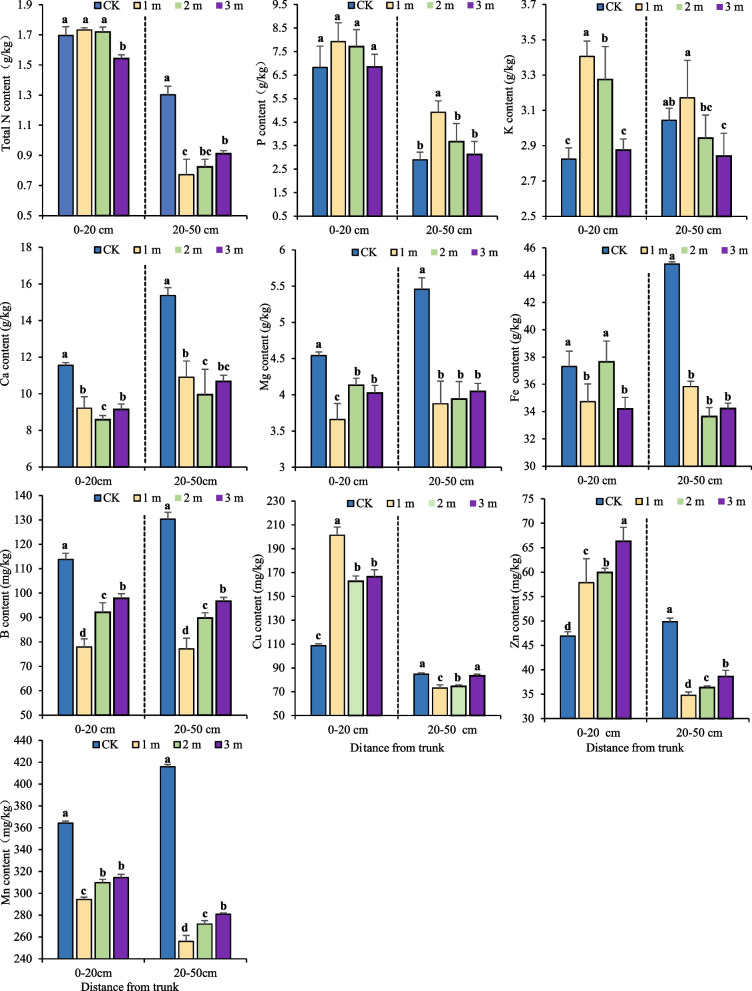
Distribution of soil nutrient elements in pear orchards. *1 m, 2 m, 3 m, representing sampling points at distances from the central stem trunk. 0–20 cm, 20–50 cm, representing the depth of the sampled soil layer

There were significant differences in terms of the distance from the center trunk and the soil depth. As shown in Fig. 5, in the 0–20 cm soil layer, the contents of N, P, K, and Cu in the soil were the highest at 1 m from the trunk, at 1.73 g·kg^−1^ and 3.41 g·kg^−1^ and 201.34 mg·kg^−1^, respectively. The contents of Ca, Mg, B, and Mn in the blank area without tree planting were the highest, at 11.55 g·kg^−1^, 4.54 g·kg^−1^, 113.76 mg·kg^−1^ and 364.38 mg·kg^−1^, respectively, indicating that the demand for these elements was greater for the pear trees during the growth process. The Zn content increased with increasing distance from the trunk, and the Fe content increased to 37.75 g·kg^−1^ at a distance from the trunk of 2 m. The contents of N and Fe were the lowest at 3 m from the trunk, at 1.54 g·kg^−1^ and 34.20 g·kg^−1^, respectively; and the contents of P, K, Cu and Zn were the lowest in the blank area without trees. In the 20–50 cm soil layer, the contents of Mg, Ca, N, Cu, B, Mn, Zn and Fe in the blank area without tree planting were the highest, at 5.46 g·kg^−1^ and 15.37 g·kg^−1^, 1.30 g·kg^−1^, 84.68 mg·kg^−1^, 130.20 mg·kg^−1^, 415.75 mg·kg^−1^, 49.82 mg·kg^−1^, and 44.81 g·kg^−1^, respectively. The potassium (K) content was highest at 1 m from the trunk (3.41 g·kg^−1^). The phosphorus (P) content was highest at 1 m from the trunk (2.92 g·kg^−1^), and the Cu content was highest in the uncultivated control area (84.68 mg·kg^−1^). The N and K contents were lowest at 3 m from the trunk, at 1.54 g·kg^−1^ and 2.84 g·kg^−1^, respectively. The P content was lowest in the uncultivated control area (6.82 g·kg^−1^). The Ca and Fe contents were lowest at 2 m from the trunk, at 9.94 g·kg^−1^ and 33.63 mg·kg^−1^, respectively. The Mg, B, Zn, and Mn contents were lowest at 1 m from the trunk, at 3.88 g·kg^−1^, 77.18 mg·kg^−1^, 34.78 mg·kg^−1^, and 256.04 mg·kg^−1^, respectively.

### Correlations between soil, tree nutrition, and microbial species at different ages

**Fig. 6:**
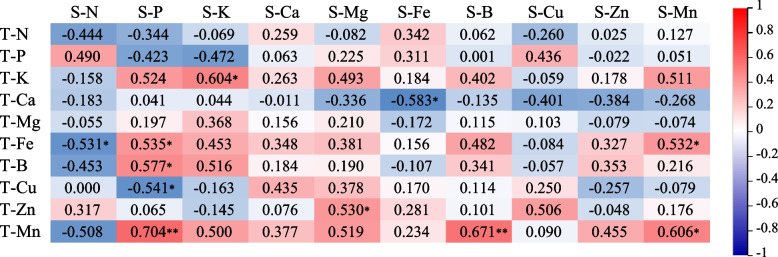
Correlations between leaf nutrition and soil nutrition. Note: S represents leaf nutrition, T represents soil nutrition. **Correlation is significant at the 0.01 level(2-tailed), * Correlation is significant at the 0.05 level (2-tailed)

To further analyze the relationship between soil nutrition and tree nutrition, the correlation between soil layer (0–20 cm) nutrition and leaf nutrition was analyzed for different age plots. Correlation analysis was performed between the average values of the soil nutrient elements and the leaf nutrient elements (Fig. 6). The results revealed that there was a significant positive correlation between soil K and leaf K, i.e., with increasing or decreasing soil K content, the K content in leaves decreased. The leaf P content was significantly positively correlated with the soil Fe, B, and Mn contents and significantly negatively correlated with the soil Cu content. The leaf N content was significantly negatively correlated with the soil Fe content, indicating that low soil iron levels can reduce the leaf nitrogen content. The leaf Fe content was significantly negatively correlated with the soil Ca content, suggesting that high-calcium soils can impair iron uptake by trees. The leaf manganese content was significantly positively correlated with both the soil iron content and the soil manganese content. The leaf boron content was strongly positively correlated with the soil manganese content.

**Fig. 7:**
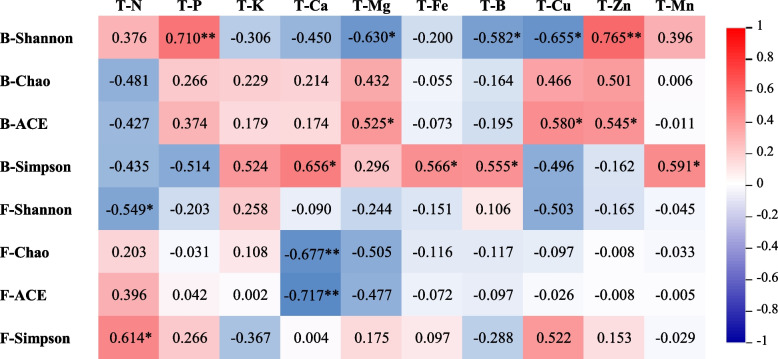
Correlations between soil nutrients and soil microorganisms. Note: B represents the bacterial community richness index, F represents the fungal community richness index, and T represents soil nutrition. ^**^Correlation is significant at the 0.01 level(2-tailed), ^*^Correlation is significant at the 0.05 level (2-tailed)

To clarify the relationship between the soil microbial community and soil nutrient distribution, a correlation analysis between nutrients and the microbial community diversity index in soil layers (0–20 cm) of different age trees was performed (Fig. 7). The bacterial Shannon index was significantly positively correlated with soil P and Zn and significantly negatively correlated with soil Mg, B, Cu and organic matter. The bacterial ACE index was significantly positively correlated with soil Mg, Cu, and Zn concentrations. The bacterial Simpson index was significantly positively correlated with soil Ca, Fe, B, and Mn concentrations. There was a significant negative correlation between the Shannon index of fungi and soil N, a very significant negative correlation between the Chao1 index and the ACE index of fungi and soil Ca, and a significant positive correlation between the Simpson index of fungi and soil N.

## Discussion

The leaf nutrient element content is among the important factors affecting the growth and development of fruit trees and fruit quality [25, 30]. A sufficient content of nutrient elements in leaves can continuously provide nutrients for fruit development, thereby improving fruit quality and increasing orchard yield [7]. Currently, various research indicates that tree age can influence fruit quality, but research on the relationship between tree age and leaf nutrient accumulation is limited [31, 32]. There are some differences in the nutrient element contents of the same variety and different ages [33]. In this study, the leaf Ca and B contents initially increased but then decreased with tree age. The Cu content of pear trees increased significantly with increasing tree age, and the contents of P, K and other elements were greatest when the trees were 65 years old. Soil analysis of plots with varying tree ages revealed that younger trees (less than 25 years old) presented relatively lower levels of nutrients, such as K, Fe, Cu, Zn, and Mn, in the soil. These findings indicate that young trees have greater nutrient requirements than older trees do, likely because of the stronger nutrient uptake capacity of young trees [18]. On the other hand, the rhizosphere microbial community of mature trees is more stable, and old trees reduce their dependence on external nutrients through optimized internal circulation and synergistic interactions with rhizosphere microorganisms [34]. Therefore, young trees require supplemental quick-release nutrients to meet microbial demands, whereas the focus should be on improving soil physicochemical properties for older trees.

Branches are the key part of the tree for the storage of nutrients, and deciduous fruit trees return their main nutrients to their branches and roots every autumn and winter [35]. The accumulation of nutrient elements in branches directly affects yield during the following year and disease resistance [36, 37]. The absorption and transport of various nutrients by pear trees occur throughout their entire growth and development period, and the content of nutrients also varies at different stages [38]. The annual demand ratios of macronutrients by the some perennials, from highest to lowest, are K, N, P, Ca, and Mg, and the annual demand ratios of micronutrients, from highest to lowest, are Fe, Zn, Mn, and Cu [39]. In this study, the tender and metabolically active parts of pear branches, such as one- and two-year-old branches, and the phloem of the main trunk accumulated relatively high levels of nutrients such as nitrogen (N) and phosphorus (P), similar to those in other woody plants [40]. The contents of elements such as N, Ca, Mg, Fe, Cu, and Mn in the xylem of the main stem were the lowest, indicating that some of these elements are less likely to accumulate in perennial above-ground tissues. Less mobile elements, such as Ca, Mn, B, Mg, and Fe, accumulated more in older perennial roots, which is consistent with previous findings [41, 42]. Furthermore, their contents in long-term pear orchard soil were generally lower than those in uncultivated soil, suggesting that while roots absorb these elements, they are difficult to transport within trees and tend to be stored in older roots. Therefore, foliar application is recommended for supplying these elements during pear tree fertilization. In this study, the concentrations of nutrients such as Ca, Mg, Be, and Mn in soils at various distances from the central trunk were significantly lower than those in the control soil. These findings indicate that pear roots absorb these elements in greater quantities, but cultivation practices often fail to adequately supplement them.

Soil is the foundation of pear production, as the pH level, organic matter content, total nitrogen, alkali-hydrolysable nitrogen, available phosphorus, and available potassium in pear orchard soil directly impact pear tree growth and fruit quality [25]. Many studies have shown that there are significant differences in soil nutrient contents across different planting years, and certain patterns are observed with increasing planting duration [43]. When managing pear orchards of different ages, the focus should be on the abundance and deficiency of microelements [44, 45]. This study revealed that the contents of macronutrients such as K and Mg in the soil tended to first increase but then decrease with increasing tree age. As trees age, changes in nutrient requirements, soil acidification, and cumulative effects from long-term cultivation cause soil levels of macronutrients such as potassium and magnesium to initially increase before declining [43, 46]. The boron content in soil tends to increase initially but then decrease with tree age, possibly because of increased boron demand in the fruit during the high-yield period of mature trees, leading to a net reduction in the amount of boron in the soil [47]. In the soils of the pear orchard with older trees, the Cu content in the 0–20 cm soil layer increased significantly. Compared with that in the soil without pear trees, the amount of Cu in the soils of the pear orchard was greater. Cu is an essential micronutrient for plants, with very low cellular requirements [48], but the increased accumulation of Cu in pear orchard soil might be related to the long-term use of copper preparations as fungicides [49].

Soil microorganisms are vital components of soil ecosystems and can not only increase the content of soil organic matter but also promote the uptake of mineral elements by trees [50]. The diversity and richness of soil microbial communities reflect dynamic changes in the overall community, which is highly important for the normal growth and development of fruit trees [20]. The results of this study indicate that soil microorganisms are dominated by bacteria, with bacterial communities being primarily dominated by the phyla Proteobacteria, Actinobacteria, Acidobacteria, and Chloroflexi, suggesting that bacterial communities in soils with different tree species may vary [51, 52]. Soil microbial communities directly influence nutrient accumulation and the sensory quality of pears by regulating the cycling of key nutrients such as nitrogen and carbon [53]. In this study, the root distribution of pear trees of different ages, the physicochemical properties of the soil, and the nutrient and element contents differed, which affected the soil microbial communities. Previous studies have shown that increasing tree age significantly increases fungal diversity but suppresses bacterial diversity [54]. On the other hand, the diversity of rhizosphere microorganisms in specific habitats may remain relatively stable and unaffected by tree age [55]. With respect to changes in microbial community structure and function, the abundance of the bacterial phylum Proteobacteria increased steadily with tree age, and the fungal community structure showed more pronounced differentiation with age, particularly between saprophytic and symbiotic functional groups [54, 56]. This study revealed that the Shannon index of the soil bacterial community was highly significantly positively correlated with soil P and Zn and significantly negatively correlated with soil Mg, B, and Cu. The fungal Chao index and ACE index were highly significantly negatively correlated with the soil Ca content, which may be related to the excessive application of calcium fertilizer and excessive Ca content in the soil. While this study has identified correlations between specific mineral nutrients and microbial communities in pear orchards, the underlying mechanisms driving these microbial responses remain to be elucidated.

## Conclusion

As pear orchards age, the contents of nutrients and microbial communities exhibit certain patterns. Specifically, the copper (Cu) content in tree tissues increases with tree age, whereas the abundance of Actinobacteria and Acidobacteria decreases with increasing tree age. Nutrient elements such as Ca, Mn, B, Mg, and Fe, which are difficult to transport, are best applied through foliar fertilization. This study clarifies the nutrient element requirements of pear orchards of different ages, providing data support for guiding rational fertilization practices.

## Supplementary Information

Supplementary Material 1.

Supplementary Material 2.

Supplementary Material 3.

## Data Availability

All data and materials are presented in the main paper and supplementary information files.

## Abbreviation

ACE: Bundance-based Coverage Estimator

## References

[1] Zhang S, Xie Z. Current status, trends, main problems and the suggestions on development of pear industry in China. J Fruit Sci. 2019;36:1067–72.

[2] Liang S, Zhao J. Analysis of economic development and contributions of Hebei’s pear fruit industry. China Fruit. 2022;12:68–73.

[3] Tian Y, Du E, Tang Y, Xia N. Distinct seasonality of nutrients in twigs and leaves of temperate trees. Tree Physiology. 2025;45(3):tpaf014. 39883083 10.1093/treephys/tpaf014

[4] Meller S, Frossard E, Luster J. Phosphorus allocation to leaves of beech saplings reacts to soil phosphorus availability. Front Plant Sci. 2019;10:744. 31244871 10.3389/fpls.2019.00744 PMC6563415

[5] Quyen NK, Dang LV, Ngoc NP, Phuong Thao PT, Hung NN. Determination of nutritional sufficiency ranges for pomelo (*Citrus grandis* Osbeck) grown on alluvial soils using DRIS. PLoS ONE. 2024;19(10):e0312231. 39413091 10.1371/journal.pone.0312231 PMC11482690

[6] Ferrández-Cámara M, Martínez-Nicolás JJ, Alfosea-Simón M, Cámara-Zapata JM, Melgarejo Moreno P, García-Sánchez F. Estimation of diagnosis and recommendation integrated system (DRIS), compositional nutrient diagnosis (CND) and range of normality (RN) norms for mineral diagnosis of almonds trees in Spain. Horticulturae. 2021;7(11):481.

[7] Osvalde A, Karlsons A, Cekstere G, Āboliņa L. Leaf nutrient status of commercially grown strawberries in Latvia, 2014–2022: a possible yield-limiting factor. Plants. 2023;12:945. 36840293 10.3390/plants12040945 PMC9963533

[8] Lombardo S, Restuccia A, Abbate C, Anastasi U, Fontanazza S, Scavo A, et al. *Trifolium subterraneum* cover cropping for improving the nutritional status of a Mediterranean apricot orchard. J Sci Food Agric. 2021;101(9):3767–77. 33300619 10.1002/jsfa.11009

[9] Almutairi KF, Abdel-Sattar M, Mahdy AM, El-Mahrouky MA. Co-application of mineral and organic fertilizers under deficit irrigation improves the fruit quality of the Wonderful pomegranate. PeerJ. 2021;9:e11328. 34046255 10.7717/peerj.11328 PMC8139271

[10] Falchi R, Bonghi C, Drincovich MF, Famiani F, Lara MV, Walker RP, et al. Sugar metabolism in stone fruit: source-sink relationships and environmental and agronomical effects. Front Plant Sci. 2020;11:573982. 33281843 10.3389/fpls.2020.573982 PMC7691294

[11] Pushpavathi Y, Satisha J, Satisha GC, Shivashankara KS, Reddy ML, Sriram S. Influence of different sources and methods of potassium application on the quality of grapes cv. Sharad Seedless (*Vitis vinifera* L.). Curr Sci. 2020;118(4):639–43.

[12] Bhagat R, Walia SS, Dheri GS, Singh G, Sharma K. Pear (*Pyrus communis*)-based agroecosystem improves soil nutrient dynamics, microbial biomass enzymatic activity farm productivity and profitability. Sci Hortic. 2024;336:113398.

[13] Sorrenti G, Toselli M, Marangoni B. Use of compost to manage Fe nutrition of pear trees grown in calcareous soil. Sci Hortic. 2012;136:87–94.

[14] Curetti M, Sánchez E, Tagliavini M, Gioacchini P. Foliar-applied urea at bloom improves early fruit growth and nitrogen status of spur leaves in pear trees, cv. Williams Bon Chretien. Sci Hortic. 2013;150:16–21.

[15] Tsai HH, Schmidt W. One way. Or another? Iron uptake in plants. New Phytol. 2017;214(2):500–5. 28205242 10.1111/nph.14477

[16] Gao C, Li C, Zhang L, Guo H, Li Q, Kou Z, et al. The influence of soil depth and tree age on soil enzyme activities and stoichiometry in apple orchards. Appl Soil Ecol. 2024;202:105600.

[17] Neto C, Carranca C, Clemente J, de Varennes A. Nitrogen distribution, remobilization and re-cycling in young orchard of non-bearing ‘Rocha’ pear trees. Sci Hortic. 2008;118(4):299–307.

[18] Fan A, Jin S, Tan Y, Huan W, Chen W, Wang X, et al. Nutrient recycling and utilization of *Torreya grandis* “Merrillii” along an age gradient. Front Plant Sci. 2025;16:1566140. 40313724 10.3389/fpls.2025.1566140 PMC12044428

[19] Ajeethan N, Ali S, Fuller KD, Abbey L, Yurgel SN. Apple root microbiome as indicator of plant adaptation to apple replant diseased soils. Microorganisms. 2023;11(6):1372. 37374874 10.3390/microorganisms11061372 PMC10301482

[20] Matthews KE, Breed MF, Stirling E, Macdonald LM, Cavagnaro TR. Comparing apples and apples; evaluating the impact of conventional and organic management on the soil microbial communities of apple orchards. Appl Soil Ecol. 2025;215:106470.

[21] Park H, Kim K, Walitang DI, Sayyed R, Sa T. Shifts in soil bacterial community composition of jujube orchard influenced by organic fertilizer amendment. J Microbiol Biotechnol. 2024;34(12):2539–46. 39628327 10.4014/jmb.2406.06037 PMC11729348

[22] Debenport Spencer J, Assigbetse K, Bayala R, Chapuis-Lardy L, Dick Richard P, McSpadden Gardener Brian B. Association of shifting populations in the root zone microbiome of millet with enhanced crop productivity in the Sahel Region (Africa). Applied and Environmental Microbiology. 2015;81(8):2841–51. 10.1128/AEM.04122-14 PMC4375326 25681183

[23] Yao S, Merwin IA, Abawi GS, Thies JE. Soil fumigation and compost amendment alter soil microbial community composition but do not improve tree growth or yield in an apple replant site. Soil Biol Biochem. 2006;38(3):587–99.

[24] Elzobair KA, Stromberger ME, Ippolito JA, Lentz RD. Contrasting effects of biochar versus manure on soil microbial communities and enzyme activities in an Aridisol. Chemosphere. 2016;142:145–52. 26138708 10.1016/j.chemosphere.2015.06.044

[25] Sun M, Zhao Y, Liang Z, Wu Y, Du R, Liu J, et al. Soil, leaf and fruit nutrient data for pear orchards located in the Circum-Bohai Bay and Loess Plateau regions. Sci Data. 2023;10:88. 36774437 10.1038/s41597-023-01999-2 PMC9922307

[26] Sete PB, Comin JJ, Nara Ciotta M, Almeida Salume J, Thewes F, Brackmann A, et al. Nitrogen fertilization affects yield and fruit quality in pear. Sci Hortic. 2019;258:108782.

[27] Miller RO. Nitric-perchloric acid wet digestion in an open vessel. In: Kalra YP, editor. Handbook of reference methods for plant analysis. Boca Raton: CRC Press; 1997. p. 57–61.

[28] Dey S, Tribedi P. Microbial functional diversity plays an important role in the degradation of polyhydroxybutyrate (PHB) in soil. 3 Biotech. 2018;8(3):171. 29556425 10.1007/s13205-018-1201-7 PMC5845049

[29] Gonzalez-Escobedo R, Briones-Roblero CI, López MF, Rivera-Orduña FN, Zúñiga G. Changes in the microbial community of *Pinus arizonica* saplings after being colonized by the bark beetle *Dendroctonus rhizophagus* (Curculionidae: Scolytinae). Microb Ecol. 2019;78(1):102–12. 30349964 10.1007/s00248-018-1274-1

[30] Dris R, Niskanen R, Fallahi E. Relationships between leaf and fruit minerals and fruit quality attributes of apples grown under northern conditions. J Plant Nutr. 1999;22(12):1839–51.

[31] Khalid S, Malik AU, Saleem BA, Khan AS, Khalid MS, Amin M. Tree age and canopy position affect rind quality, fruit quality and rind nutrient content of ‘Kinnow’ mandarin (*Citrus nobilis* Lour×*Citrus deliciosa* Tenora). Sci Hortic. 2012;135:137–44.

[32] Meena NK, Asrey R. Tree age affects physicochemical, functional quality and storability of Amrapali mango (*Mangifera indica* L.) fruits. J Sci Food Agric. 2018;98(9):3255–62. 29230820 10.1002/jsfa.8828

[33] Zhang H, Sun M, Wen Y, Tong R, Wang G, Wu Q, et al. The effects of stand age on leaf N:P cannot be neglected: a global synthesis. For Ecol Manage. 2022;518:120294.

[34] Lan X, Ning Z, Jia Y, Lin W, Xiao E, Cheng Q, et al. The rhizosphere microbiome reduces the uptake of arsenic and tungsten by *Blechnum orientale* by increasing nutrient cycling in historical tungsten mining area soils. Sci Total Environ. 2024;924:171429. 38442750 10.1016/j.scitotenv.2024.171429

[35] Kurita Y, Baba K, Ohnishi M, Matsubara R, Kosuge K, Anegawa A, et al. Inositol hexakis phosphate is the seasonal phosphorus reservoir in the deciduous woody plant *Populus alba* L. Plant Cell Physiol. 2017;58(9):1477–85. 28922751 10.1093/pcp/pcx106

[36] Le Roncé I, Dardevet E, Venner S, Schönbeck L, Gessler A, Chuine I, et al. Reproduction alternation in trees: testing the resource depletion hypothesis using experimental fruit removal in *Quercus ilex*. Tree Physiol. 2023;43(6):952–64. 36892403 10.1093/treephys/tpad025

[37] González de Andrés E, Gazol A, Querejeta JI, Igual JM, Colangelo M, Sánchez-Salguero R, Linares JC, Camarero JJ. The role of nutritional impairment in carbon-water balance of silver fir drought-induced dieback. Global Change Biology. 2022;28(14):4439–58. 10.1111/gcb.16170 PMC9540818 35320604

[38] Colpaert B, Steppe K, Gomand A, Vanhoutte B, Remy S, Boeckx P. Experimental approach to assess fertilizer nitrogen use, distribution, and loss in pear fruit trees. Plant Physiol Biochem. 2021;165:207–16. 34052682 10.1016/j.plaphy.2021.05.019

[39] Liao W, Wang H, Fan H, Chen J, Yin L, Cai X, et al. Nutrient and biomass dynamics for dual-organ yield in turmeric (*Curcuma longa* L.). PeerJ. 2025;13:e19933. 40895071 10.7717/peerj.19933 PMC12399080

[40] Lu S, Xv J, Gong Y, Gong W, Hui W, Qiu J, et al. Seasonal changes of mineral nutrient absorption and allocation in the branch and leaf of *Zanthoxylum bungeanum* “Hanyuan” during the fruit development. Front Plant Sci. 2024;15:1484762. 39619850 10.3389/fpls.2024.1484762 PMC11606090

[41] Khan FW, Khan R, Ahmed N, Saddique M, Taj R, Abdullaev S, et al. Evaluation of laser-induced breakdown spectroscopy for nutritional and trace elemental mapping in Otostegia limbata medicinal plant. Spectrochim Acta Part A Mol Biomol Spectrosc. 2026;344(Pt 1):126645. 10.1016/j.saa.2025.126645 40627936

[42] Pędziwiatr A, Kierczak J, Potysz A, Pietranik A. Effect of soil additives on biogeochemistry of ultramafic soils-an experimental approach with *Brassica napus* L. Environ Monit Assess. 2024;196(8):744. 39017939 10.1007/s10661-024-12897-4 PMC11254991

[43] Tao L, Zhang C, Ying Z, Xiong Z, Vaisman HS, Wang C, et al. Long-term continuous mono-cropping of *Macadamia integrifolia* greatly affects soil physicochemical properties, rhizospheric bacterial diversity, and metabolite contents. Front Microbiol. 2022;13:952092. 36274682 10.3389/fmicb.2022.952092 PMC9582743

[44] Álvarez-Fernández A, Melgar JC, Abadía J, Abadía A. Effects of moderate and severe iron deficiency chlorosis on fruit yield, appearance and composition in pear (*Pyrus communis* L.) and peach (*Prunus persica* (L.) Batsch). Environ Exp Bot. 2011;71(2):280–6.

[45] Buwalda JG, Meekings JS. Seasonal accumulation of mineral nutrients in leaves and fruit of Japanese pear (*Pyrus serotina* Rehd.). Sci Hortic. 1990;41(3):209–22.

[46] Li G, Liu J, Tian Y, Chen H, Ren H. Investigation and analysis of rhizosphere soil of bayberry-decline-disease plants in China. Plants. 2022;11:3394. 36501433 10.3390/plants11233394 PMC9740188

[47] Botelho RV, Müller MML, Umburanas RC, Laconski JMO, Terra MM. Boron in fruit crops: plant physiology, deficiency, toxicity, and sources for fertilization. In: Aftab T, Landi M, Papadakis IE, Araniti F, Brown PH, editors. Boron in Plants and Agriculture. New York: Academic Press; 2022. p. 29–50.

[48] Printz B, Lutts S, Hausman JF, Sergeant K. Copper trafficking in plants and its implication on cell wall dynamics. Front Plant Sci. 2016;7:601. 27200069 10.3389/fpls.2016.00601 PMC4859090

[49] Brunetto G, Bastos de Melo GW, Terzano R, Del Buono D, Astolfi S, Tomasi N, Pii Y, Mimmo T, Cesco S. Copper accumulation in vineyard soils: Rhizosphere processes and agronomic practices to limit its toxicity. Chemosphere. 2016; 162:293–307. 10.1016/j.chemosphere.2016.07.104 27513550

[50] Pradhan N, Singh S, Saxena G, Pradhan N, Koul M, Kharkwal AC, et al. A review on microbe-mineral transformations and their impact on plant growth. Front Microbiol. 2025;16:1549022. 40822401 10.3389/fmicb.2025.1549022 PMC12350396

[51] Longa CMO, Antonielli L, Bozza E, Sicher C, Pertot I, Perazzolli M. Plant organ and sampling time point determine the taxonomic structure of microbial communities associated to apple plants in the orchard environment. Microbiol Res. 2022;258:126991. 35219161 10.1016/j.micres.2022.126991

[52] Wang L, Ye X, Shen Z, Zhang Y, Lin J. Identifying the effects of cropping with different pear cultivars on microbial community composition and networks in orchard soils. Environ Sci Pollut Res Int. 2023;30(24):66157–69. 37097572 10.1007/s11356-023-26944-z

[53] Shi C, Wang X, Jiang S, Xu J, Luo J. Investigating the impact of long-term bristlegrass coverage on rhizosphere microbiota, soil metabolites, and carbon-nitrogen dynamics for pear agronomic traits in orchards. Front Microbiol. 2024;15:1461254. 39301192 10.3389/fmicb.2024.1461254 PMC11411186

[54] Liu X, Hou L, Ding C, Su X, Zhang W, Pang Z, et al. Effects of stand age and soil microbial communities on soil respiration throughout the growth cycle of poplar plantations in northeastern China. Front Microbiol. 2024;15:1477571. 39664057 10.3389/fmicb.2024.1477571 PMC11631584

[55] Yuan G, Zheng Y, Sun X. Unveiling microbial dynamics: how forest aging shapes the microbial communities of *Pinus massoniana*. Ecol Evol. 2025;15(3):e71132. 40071151 10.1002/ece3.71132 PMC11896641

[56] Zhang J, Liu Q, Wang D, Zhang Z. Soil microbial community, soil quality, and productivity along a chronosequence of *Larix principis-rupprechtii* forests. Plants. 2023;12(16):2913. 37631125 10.3390/plants12162913 PMC10458017

