# Supplementary material for: Comprehensive analysis of nutrient partitioning and microbial communities in pear orchards: effects of tree age and spatial heterogeneity
Source: BMC Plant Biol. 2025 Dec 2;25:1673. doi: 10.1186/s12870-025-07704-2 (PMC12673680; doi:10.1186/s12870-025-07704-2)
Supplement: Supplementary file 1 — Supplementary Material 1. [file 12870_2025_7704_MOESM1_ESM.docx]

**Soil Microbial Community Diversity Indices**

Detailed Explanation of Calculation Methods

H

**Shannon Index (H')**

The Shannon Index, also known as the Shannon-Wiener Index or Shannon Diversity Index, is a measure of species diversity that considers both species richness and evenness.

H' = -∑(p_i_ × ln(p_i_))

**Variables:**

- **H'**: Shannon diversity index
- **p_i_**: Proportion of individuals belonging to the i-th species (n_i_/N)
- **n_i_**: Number of individuals of species i
- **N**: Total number of individuals in the sample
- **ln**: Natural logarithm

The Shannon index increases as both the richness (number of species) and the evenness (distribution of individuals among species) increase. Higher values indicate greater diversity.

In microbial ecology, this index is calculated based on the relative abundance of different microbial taxa (usually at the species or OTU level) derived from sequencing data.

D

**Simpson Index (D)**

The Simpson Index measures the probability that two individuals randomly selected from a sample will belong to the same species. It gives more weight to common species.

D = ∑(p_i_)^2^

**Variables:**

- **D**: Simpson diversity index
- **p_i_**: Proportion of individuals belonging to the i-th species (n_i_/N)

The Simpson index ranges from 0 to 1, where 0 represents infinite diversity and 1 represents no diversity. Sometimes the index is expressed as 1-D or 1/D to make higher values indicate greater diversity.

In microbial studies, the Simpson index is often reported as 1-D to maintain the intuitive relationship where higher values mean greater diversity.

ACE

**ACE Index**

The ACE (Abundance-based Coverage Estimator) index is a non-parametric method for estimating the total species richness in a community, particularly useful for communities with many rare species.

S_ACE_ = S_abund_ + S_rare_/C_ACE_ + (F_1_/C_ACE_)γ^2^_ACE_

**Variables:**

- **S_ACE_**: Estimated total species richness
- **S_abund_**: Number of abundant species (with more than 10 individuals)
- **S_rare_**: Number of rare species (with 10 or fewer individuals)
- **C_ACE_**: Sample coverage estimate = 1 - F_1_/N_rare_
- **F_1_**: Number of singleton species (species with exactly one individual)
- **N_rare_**: Total number of individuals in rare species
- **γ^2^_ACE_**: Coefficient of variation for rare species

The ACE index is particularly useful in microbial ecology where sequencing depth may not capture all rare species in a community. It provides an estimate of the true species richness by accounting for unseen species.

Chao

**Chao Index**

The Chao index is another non-parametric estimator of species richness, particularly focused on accounting for unseen species based on the number of rare species in a sample.

S_Chao1_ = S_obs_ + (F_1_^2^)/(2×F_2_)

**Variables:**

- **S_Chao1_**: Estimated total species richness
- **S_obs_**: Number of observed species
- **F_1_**: Number of singleton species (species with exactly one individual)
- **F_2_**: Number of doubleton species (species with exactly two individuals)

The Chao1 index is one of the most widely used richness estimators in microbial ecology. It is based on the concept that rare species (particularly singletons and doubletons) provide information about undetected species.

This estimator tends to perform well with communities that have many rare species and is less biased than other estimators when sample sizes are small.
